# Neighborhood social capital and sleep duration: a population based cross-sectional study in a rural Japanese town

**DOI:** 10.1186/s12889-018-5204-4

**Published:** 2018-03-12

**Authors:** Thida Win, Toru Yamazaki, Koji Kanda, Kazuo Tajima, Shigeru Sokejima

**Affiliations:** 1 Department of Public Health and Occupational Medicine, Mie University Graduate School of Medicine, 2-174 Edobashi, Tsu-shi, Mie 514-8507 Japan; 2 Epidemiology Centre for Disease Control and Prevention, Mie University Hospital, Tsu, Mie Japan; 3 Japan International Cooperation Agency, Tokyo, Japan

**Keywords:** Neighborhood social capital, Gender, Health behavior, Sleep duration

## Abstract

**Background:**

Studies on social capital and health outcomes have become common, but the relationship between neighborhood social capital and sleep duration by gender is still unclear. We examined the relationship between neighborhood social capital and sleep duration by gender in adults living in a rural community in Japan.

**Method:**

We conducted a cross-sectional survey of 12,321 residents aged ≥20 years in a town in Mie Prefecture in January–March 2013. Self-completed questionnaires were collected from the residents (*n* = 7782; valid participation rate, 63.2%). We used five items to assess the neighborhood social capital (Cronbach’s α = 0.86). We summed up the scores of each item, and then divided the participants into four groups by quartile of total scores of neighborhood social capital (lowest, low, high, and highest). Sleep duration of < 7 h/day was defined as insufficient sleep duration according to previous studies. To adjust for potential confounders, we performed a multiple log-binominal regression analysis and estimated the prevalence ratios (PRs) and 95% confidence intervals (CIs) for insufficient sleep.

**Results:**

Overall 42% of the men and 45% of the women had insufficient sleep. In the men, the lowest group of neighborhood social capital presented a 22% higher prevalence of insufficient sleep (PR 1.22; 95% CIs 1.08–1.38) compared to the highest group of neighborhood social capital. Similarly the low group of neighborhood social capital and the high group of neighborhood social capital had 20 and 19% higher prevalence of insufficient sleep (PR 1.20; 95% CIs 1.06–1.36; PR 1.19; 95% CIs 1.06–1.34, respectively) compared to the highest group of neighborhood social capital. For women there was no significant association between neighborhood social capital and insufficient sleep after controlling for all potential confounders.

**Conclusion:**

Having lower neighborhood social capital was associated with insufficient sleep among Japanese adults, particularly in the men. This suggests that the context of neighborhood social capital by gender should be considered to promote healthier behaviors with regard to getting enough sleep.

**Electronic supplementary material:**

The online version of this article (10.1186/s12889-018-5204-4) contains supplementary material, which is available to authorized users.

## Background

Studies on social capital and health outcomes have become much more common, and social capital is considered an important determinant of health [1]. ‘Social capital’ is defined as “resources composed of or derived from trust, and/or norms (especially reciprocity), and/or networks, which facilitate collective actions” [2]. The resources generated by a community or neighborhood are referred to as “neighborhood social capital” (NSC) [3]. A highly cohesive community is crucial in attempts to promote healthier behaviors. For example, a well-connected neighborhood enables its residents to easily distribute information on health-promoting behaviors [4]. A supportive neighborhood also advocates healthier behaviors through psychosocial or stress coping mechanisms [5]. Moreover, a community with informal social control enhances the maintenance of healthy behavioral norms or the deterrence of risky behaviors among neighbors [6].

Several studies have shown that health-related behaviors such as smoking, alcohol drinking, physical activity, and sleep are important mediators between NSC and health outcomes [3, 7, 8]. However few studies described the direct relationship between NSC and sleep disorders [9, 10]. In addition, NSC differs between the genders in terms of social networks and opportunities to invest in social capital [11]. For instance, Elisabetta Addis and Majlinda Joxhe presented that men and women differ in network structure and accumulation of social capital which is not only reflecting the basic biological sex but also the diverse constructs of behavior, attitude and norms of the society [12]. The gender gap is largely reflected in the way that the day-to-day activities of men and women differ, and studies of social capital should therefore consider the gender differences [13]. Previous studies also showed inconsistent gender differences in the relationship between social capital and health outcomes. For example, a study in Japan showed that the women having stronger bridging social capital were more likely to have the depressive mood but the association was not found in the men [14]. With the greater magnitude for women health than for men, M. Stafford et al. described the presence of gender role in the neighborhood characteristics such as trust, integration into wider society etc. [15]. Conversely, in Russia, men with increased formal and informal social participation reported poorer health than who did not whereas no such significant relationship were found in women [16]. Therefore it is important enough to detect the role of gender in studies of social capital and health outcomes. However, there has been relatively little research into gender differences in NSC and sleep disorders.

Sleep disorders have been a key public health issue. Sleep problems are a major challenge in developed countries, and sleeping disturbances were found in one out of five people in Japan [17, 18]. For instance, the recommended sleep duration for a normal adult person is 7–9 h (hrs) according to the U.S. National Sleep Foundation [19]. According to a report by the Organization for Economic Co-operation and Development (OECD), average sleep duration of the OECD countries was 8 h and 22 min (min), but Japan had the second-least average sleep duration (7 h 43 min) among the OECD countries [20]. Japanese Statistics Bureau also reported that sleeping time in Japan for both sexes was declining over past two decades [21]. Although both short and long sleeping times can cause health problems, several studies reported that short sleep duration was rather related to adverse health outcomes [22–25]. Particularly in Japan, short duration of sleep is the most frequent problem because of long working hours and less sleeping time [26]. The prevalence of short sleeping time in Japanese was ranging from 47.6 to 51.5% although the definitions of short duration of sleep differed among studies [26–28]. Sleep duration < 7 h was especially associated with mortality and metabolic risk factors in Japan [25, 28, 29].

Moreover, insufficient sleep induces poor health through several triggering factors [30–32]. For example, sleep disturbances induced by multiple stressors can cause mental illnesses [33, 34]. Socio-environmental factors also have enormous effects on insufficient sleep [35–37]. Gender differences in sleep are also very important because of their different attribution to biological, psychosocial and cultural influences [38]. For example the brain activity of men and women differs before and after sleep [39] and this underlying biological difference is worth to recognize in sleep studies. Moreover Sarah A. Burgard pointed out that women had disproportionate burden of interrupted sleep for caregiving because of differences in social roles and work-family responsibilities than men do [40]. Previous studies suggested that a sleep study in relation to health outcomes should be conducted by genders because there were not any consistent differences in genders across the studies [41, 42]. In addition, gender differences can exist in the relation between sleep and socio-environmental factors such as neighborhood disadvantages or neighborhood social capital [10, 43]. However, the evidence is limited for the association of NSC in relationship to insufficient sleep in accord with gender. In the present study we thus examined the association between NSC and insufficient sleep by gender in a general population of Japanese adults.

## Methods

### Data collection

We conducted a population-based cross-sectional survey in a rural town located in eastern Mie Prefecture, where a total of 15,280 residents lived at the time of our survey. The survey was conducted in 2013 from January to March using self-completed questionnaire, targeting 12,321 residents aged ≥20 years. The questionnaire asked the respondent about his or her socioeconomic status, lifestyle, sleep hours, work hours, social capital, self-rated health, and non-communicable diseases (NCDs). Of the target population, 7782 residents gave consent to use their information in this study, and the valid participation rate was 63.2%. Of the residents, the respondents who did not answer the age and gender questions (0.78%, *n* = 61) were regarded as unreliable and were excluded from the analysis. A final total of 7721 respondents were analyzed. The study protocol was approved by the Ethical Committee of the Medical Department of Mie University, Japan (Approved number: 1268). Written informed consent was obtained from all participants. The study was conducted in accordance with the Helsinki Declaration.

### Measurement of neighborhood social capital (NSC)

In our study, the definition of NSC involved cognitive and structural dimensions. Neighborhood social capital was assessed by the individual respondent’s perception of social capital in his or her neighborhood reflecting the feeling of fellowship, emotional social support, norms of reciprocity, perceived neighborhood trust, and social activity or co-operation. The NSC questionnaire was derived from the questionnaire of Cabinet Office, Government of Japan (COJ), 2003 [44]. The respondents were required to answer the following five items: (1) I feel myself as a member of neighbors (feeling of fellowship). (2) I have someone in my neighborhood to consult when I need to talk to someone (emotional social support). (3) I like someone in my neighborhood who has the same idea as me (reciprocity norm). (4) I think people in my neighborhood trust each other (perceived neighborhood trust). (5) I and my neighbors perform social activities together for the betterment of the neighborhood (social activity or co-operation) [45–48].

These five items were measured on a five-point Likert scale: strongly disagree, disagree, neither, agree, and strongly agree. We summed up the scores of each item on the questionnaire, and then divided the participants into four groups by quartiles of total NSC score, where the 1st quartile was defined as the ‘lowest’ NSC, the 2nd quartile was defined as the ‘low’ NSC, the 3rd quartile defined as the ‘high’ NSC, and the 4th quartile defined as the ‘highest’ NSC.

### Sleeping time

The participants were asked, ‘In the past 1 month, on average, how many hours of actual sleep do you generally get in a day? According to Kripke et al. [49], sleeping times < 2 h and > 16 h (0.35%) were considered unreliable and invalid. In the present study we thus coded these sleep durations as missing values. Based on our review of relevant studies [8, 19, 25, 50, 51], we defined the sleep duration of 7 h per day as the optimum number of hours of sleep for adults. In accordance with a previous study in Japan [8], we dichotomized the respondents’ sleep durations into ≥7 h per day and < 7 h per day. Insufficient sleep was considered a sleep duration of < 7 h per day.

### Covariates

We selected the following as potential confounders in our study: age, gender, educational attainment, occupation, annual household income, smoking, alcohol drinking, physical activity, body mass index (BMI), and presence of non-communicable disease (NCD). Age was grouped into the following four categories: 20–39, 40–59, 60–79, and ≥80 years. Educational attainment was defined as low (elementary school, junior and senior high school), medium (college and vocational schools), and high (university, Master’s degree and above). The respondents’ occupations were categorized into the following three groups: blue collar, white collar, and others (i.e., housewives and students) [52].

Annual household income was collapsed into five categories: < 2 million yen, 2-million to less than 4 million yen, 4-million to less than 6 million yen, 6-million to less than 8 million yen, and ≥8 million yen [47]. For smoking, the following three groups were created: smoker, ex-smoker and non-smoker. Alcohol drinking was also classified into daily drinker, occasional drinker and non-drinker. With regard to physical activity, ≥150 min per week of physical activity was regarded as physically active, and < 150 min per week was physically inactive according to the WHO recommendation for adults [53].

BMI (kg/m^2^) was also classified by the following WHO recommendation [54]: underweight (< 18.50 BMI), normal (18.50–24.99), overweight (25.00–29.99) and obese (≥30). If a respondent reported having any one of the following non-communicable diseases (NCD) (stroke, hypertension, diabetes mellitus, myocardial infarction, allergy, asthma, and cancer), he or she was considered to have an NCD in this study.

### Statistical analysis

We conducted an internal reliability of NSC questions by using Cronbach’s α. For the evaluation of a high degree of correlation among independent variables, we used related statistics such as variance inflation factor (VIF) and tolerance value (TOL) [55]. By considering the importance and inconsistent results of gender differences in NSC and sleep disorders from the previous studies [56–58], we carried out a gender-stratified analysis. We calculated frequency distribution to describe the basic characteristics of the participants. Bivariate analysis of the chi-square test for independency and analysis of variances was done to assess the differences in characteristics among NSC groups. We also conducted the interaction tests for socioeconomic factors in the relationship between NSC and insufficient sleep to find the effect modification by adding the interaction term in the log-binomial model. For missing data, the fully conditional specification (FCS) method of the multiple imputation (MI) procedure was used to replace the missing values, and it was performed separately for the men and the women [59].

A total of 10 imputed datasets by gender were created for the analysis. The variables in the imputation model were sleep duration, NSC, age, education, occupation, annual household income, smoking, alcohol drinking, physical activity, BMI and NCD. We performed a multiple regression using log-binomial model with the command, “proc genmod” and “log link” to calculate the prevalence ratios by analyzing the effects of covariates concerning the association between NSC and insufficient sleep [60]. The analysis was conducted on each imputed data set, and we then pooled the results of 10 data sets for each gender, adjusting the standard errors appropriately. In addition we performed the subgroup analysis for the working age group (20–69) by considering the actual retirement age [61]. Statistical significance was set at a two-sided *P-*value < 0.05. We used SAS ver. 9.4 software (SAS Institute, Cary, NC, USA) for all of the statistical analyses.

## Results

The internal consistency reliability (Cronbach’s α) for the NSC questions was acceptably high at 0.86. There was no significant correlation among the independent variables. Overall, 43.6% (3370/7721) of the respondents experienced the insufficient sleep, with the mean duration of sleeping hours 6.8 (SD = 1.34 h). The mean sleep duration was not significantly different between the men (6.8 h) and the women (6.7 h). Although social activity or cooperation in relation to insufficient sleep had significant interaction test for gender (*P = 0.009*), none of NSC groups and other dimensions were significant for gender interaction test. However we performed all the analyses stratified by gender because of the considerable biological and psychosocial roles of gender in both NSC and sleep.

Table 1 summarizes the demographic characteristics of respondents by genders. More than half of the respondents were female (*n* = 4096; 53.1%), 45.3% of the women experienced the insufficient sleep, whereas 41.7% of the men did. More than 65.0% of both the men and the women were classified as having low education, and 43.6% of the men and 31.0% of the women were blue-collar workers. Approximately 50% of the men earned ≥4 million yen/year, and the annual household income was higher in the men than the women. A total of 33.6% of the men were smokers and 21.4% were daily drinkers, whereas among the women, only 7.6% were smokers and 3.5% were daily drinkers. More than 40% of the respondents of both genders were physically inactive, and overweight or obesity was found in 24.5% of the men and 13.3% of the women. More than half of the respondents of both genders had one of the NCDs. The gender-stratified univariate analysis showed that all of the variables were significantly associated with insufficient sleep.

**Table 1:** Demographic characteristic of the respondents

	Men (*n* = 3625, 46.9%)		Women (*n* = 4096, 53.1%)	
Characteristics	No.	(%)	No.	(%)
Neighborhood social capital				
Lowest	812	(22.4)	865	(21.1)
Low	852	(23.5)	901	(22.0)
High	1021	(28.2)	1189	(29.0)
Highest	736	(20.3)	868	(21.2)
Missing	204	(5.6)	273	(6.7)
Age (years)				
20–39	935	(25.8)	1036	(25.3)
40–59	1208	(33.3)	1346	(32.9)
60–79	1198	(33.1)	1286	(31.4)
≥ 80	284	(7.8)	428	(10.5)
Educational attainment				
Low	2496	(68.9)	2774	(67.7)
Medium	329	(9.1)	904	(22.1)
High	755	(20.8)	335	(8.2)
Missing	45	(1.2)	83	(2.0)
Occupation				
Blue collar	1581	(43.6)	1270	(31.0)
White collar	1111	(30.7)	1026	(25.1)
Others	852	(23.5)	1711	(41.8)
Missing	81	(2.2)	89	(2.2)
Annual household income (Million Yen)				
< 2	105	(2.9)	174	(4.3)
2–3.99	502	(13.9)	526	(12.8)
4–5.99	602	(16.6)	572	(14.0)
6–7.99	485	(13.4)	483	(11.8)
≥ 8	708	(19.5)	657	(16.0)
Missing	1223	(33.7)	1684	(41.1)
Smoking status				
Non-smoker	1909	(52.7)	3629	(88.6)
Ex-smoker	464	(12.8)	117	(2.9)
Smoker	1217	(33.6)	313	(7.6)
Missing	35	(1.0)	37	(0.9)
Alcohol Drinking				
Non-drinker	1453	(40.1)	2971	(72.5)
Occasional drinker	1366	(37.7)	946	(23.1)
Daily drinker	775	(21.4)	142	(3.5)
Missing	31	(0.9)	37	(0.9)
Physical activity				
Active	1517	(41.9)	1490	(36.4)
Inactive	1593	(43.9)	1993	(48.7)
Missing	515	(14.2)	613	(15.0)
BMI (kg/m^2^)				
Underweight	198	(5.5)	525	(12.8)
Normal	2432	(67.1)	2818	(68.8)
Overweight	781	(21.5)	469	(11.5)
Obese	110	(3.0)	72	(1.8)
Missing	104	(2.9)	212	(5.2)
Non-communicable disease				
Present	1859	(51.3)	2175	(53.1)
Absent	1717	(47.4)	1858	(45.4)
Missing	49	(1.4)	63	(1.5)
Insufficient sleep				
Yes	1513	(41.7)	1857	(45.3)
No	1941	(53.5)	2014	(49.2)
Missing	171	(4.7)	225	(5.5)

The characteristics of the participants across the NSC groups are shown in Table 2. The NSC scores among the NSC groups were not much different between the men and the women. The proportion of insufficient sleep of the highest NSC group in the women (42.4%) was higher than that of the men (33.7%). In both genders, older participants had higher NSC than younger participants. On the other hand other characteristics of the participants such as educational attainment, occupation, annual household income, smoking status, alcohol drinking, physical activity, BMI, and NCD were not much different among the NSC groups in both genders. There were no interactions between NSC groups and socioeconomic factors in both genders, namely age (*P* = 0.96 in men and *P* = 0.40 in women), educational attainment (*P* = 0.34 in men and *P* = 0.67 in women), occupation (*P* = 0.07 in men and *P* = 0.45 in women), annual household income (*P* = 0.21 in men and *P* = 0.88 in women).

**Table 2:** Characteristic distribution of respondents among neighborhood social capital groups

	Neighborhood social capital (*n* = 7244)															
	Men (*n* = 3421)								Women (*n* = 3823)							
	Lowest		Low		High		Highest		Lowest		Low		High		Highest	
	(*n* = 812)		(*n* = 852)		(*n* = 1021)		(*n* = 736)		(*n* = 865)		(*n* = 901)		(*n* = 1189)		(*n* = 868)	
Characteristics	No.	% or SD	No.	% or SD	No.	% or SD	No.	% or SD	No.	% or SD	No.	% or SD	No.	% or SD	No.	% or SD
Neighborhood social capital scores																
Mean	10.1	(2.6)	15.1	(0.6)	18.6	(1.1)	23.0	(1.6)	10.1	(2.6)	15.1	(0.7)	18.6	(1.1)	22.9	(1.6)
Age (years)																
20–39	310	(38.2)	324	(38.0)	180	(17.6)	93	(12.6)	339	(39.2)	295	(32.7)	255	(21.5)	124	(14.3)
40–59	267	(32.9)	295	(34.6)	395	(38.7)	212	(28.8)	303	(35.0)	318	(35.3)	443	(37.3)	240	(27.7)
60–79	193	(23.8)	197	(23.1)	376	(36.8)	350	(47.6)	155	(17.9)	211	(23.4)	395	(33.2)	397	(45.7)
≥ 80	42	(5.2)	36	(4.2)	70	(6.9)	81	(11.0)	68	(7.9)	77	(8.6)	96	(8.1)	107	(12.3)
Educational attainment																
Low	532	(66.1)	555	(65.8)	686	(67.7)	566	(77.5)	505	(59.9)	603	(67.9)	819	(69.5)	637	(74.5)
Medium	82	(10.2)	96	(11.4)	88	(8.7)	55	(7.5)	227	(26.9)	212	(23.9)	261	(22.2)	174	(20.4)
High	191	(23.7)	192	(22.8)	240	(23.7)	109	(14.9)	111	(13.2)	73	(8.2)	98	(8.3)	44	(5.2)
Occupation																
Blue collar	369	(45.8)	390	(46.0)	432	(42.6)	332	(45.5)	285	(33.4)	291	(32.6)	363	(30.8)	277	(32.1)
White collar	266	(33.0)	275	(32.5)	345	(34.0)	204	(28.0)	267	(31.3)	256	(28.7)	324	(27.5)	156	(18.1)
Others	170	(21.1)	182	(21.5)	237	(23.4)	193	(26.5)	301	(35.3)	345	(38.7)	493	(41.8)	429	(49.8)
Annual household income (Million yen)																
< 2	25	(4.8)	22	(4.2)	29	(3.9)	22	(4.1)	29	(5.7)	46	(8.4)	54	(7.1)	31	(6)
2–3.99	126	(24.1)	104	(19.6)	139	(18.6)	120	(22.4)	115	(22.5)	116	(21.3)	157	(20.5)	115	(22.2)
4–5.99	137	(26.2)	129	(24.3)	176	(23.6)	141	(26.3)	120	(23.4)	117	(21.4)	191	(24.9)	127	(24.6)
6–7.99	94	(18.1)	117	(22.1)	160	(21.5)	103	(19.2)	97	(19.0)	111	(20.3)	158	(20.6)	111	(21.5)
≥ 8	141	(27.0)	158	(29.8)	242	(32.4)	150	(28.0)	151	(29.5)	156	(28.6)	206	(26.9)	133	(25.7)
Smoking status																
Non-smoker	398	(49.4)	421	(49.7)	531	(52.3)	423	(57.8)	720	(84.2)	776	(86.6)	1082	(91.3)	802	(93.4)
Ex-smoker	112	(13.9)	97	(11.5)	150	(14.8)	91	(12.4)	31	(3.6)	35	(3.9)	27	(2.3)	21	(2.4)
Smoker	295	(36.7)	329	(38.8)	335	(33.0)	218	(29.8)	104	(12.2)	85	(9.5)	76	(6.4)	36	(4.2)
Alcohol Drinking																
Non-drinker	336	(41.6)	358	(42.2)	356	(35.0)	295	(40.4)	568	(66.2)	634	(70.5)	856	(72.5)	675	(78.7)
Occasional drinker	313	(38.8)	328	(38.7)	419	(41.2)	250	(34.3)	251	(29.3)	238	(26.5)	275	(23.3)	159	(18.5)
Daily drinker	158	(19.6)	162	(19.1)	241	(23.7)	185	(25.3)	39	(4.6)	27	(3.0)	50	(4.2)	24	(2.8)
Physical activity																
Active	310	(44.2)	337	(45.7)	479	(53.2)	336	(53.0)	279	(37.7)	318	(40.1)	460	(45.0)	379	(50.7)
Inactive	392	(55.8)	401	(54.3)	421	(46.8)	298	(47.0)	461	(62.3)	475	(59.9	562	(55.0)	369	(49.3)
BMI (Kg/m^2^)																
Underweight	55	(7.0)	50	(6.0)	45	(4.5)	29	(4.0)	135	(16.5)	110	(12.9)	142	(12.5)	106	(12.6)
Normal	512	(64.8)	584	(69.9)	700	(69.8)	512	(71.4)	576	(70.4)	627	(73.4)	837	(73.7)	612	(72.9)
Overweight	188	(23.8)	178	(21.3)	226	(22.5)	158	(22.0)	88	(10.8)	100	(11.7)	131	(11.5)	113	(13.5)
Obese	35	(4.4)	24	(2.9)	32	(3.2)	18	(2.5)	19	(2.3)	17	(2.0)	25	(2.2)	9	(1.1)
Non-communicable disease:																
Present	374	(46.8)	392	(46.3)	548	(54.3)	427	(58.6)	420	(49.5)	432	(48.6)	626	(53.0)	524	(61.0)
Absent	426	(53.3)	454	(53.7)	461	(45.7)	302	(41.4)	428	(50.5)	457	(51.4)	556	(47.0)	335	(39.0)
Sleeping time																
Mean	6.7	(1.4)	6.7	(1.3)	6.8	(1.3)	7.1	(1.3)	6.6	(1.4)	6.7	(1.4)	6.7	(1.3)	6.9	(1.2)
Insufficient sleep																
Yes	385	(49.0)	392	(47.9)	446	(44.9)	238	(33.7)	429	(51.6)	435	(50.5)	568	(48.8)	352	(42.4)
No	400	(51)	427	(52.1)	548	(55.1)	468	(66.3)	403	(48.4)	426	(49.5)	595	(51.2)	479	(57.6)

Table 3 describes the results of the multiple regression with log-binomial models using the multiple imputation method. Across all models, a significant association was found between NSC and insufficient sleep after controlling for all potential confounders in the men. Among the men, the lowest NSC group had 22% higher prevalence of insufficient sleep (PR 1.22; 95% CIs 1.08–1.38) compared to the highest NSC group. Similarly, the low NSC group and the high NSC group had 20 and 19% higher prevalence of insufficient sleep (PR 1.20; 95% CIs 1.06–1.36 and PR 1.19; 95% CIs 1.06–1.34, respectively) compared to the highest NSC group. In contrast, for the women, there was no significant association between NSC and insufficient sleep after controlling for all potential confounders. Moreover similar pattern of associations were found in the men in the relationship between each dimension of NSC except the feeling of fellowship and insufficient sleep (Table 4). The subgroup analysis of the working age group also showed the significant association between NSC and insufficient sleep in both genders (see Additional file 1).

**Table 3:** Prevalence ratios of neighborhood social capital for insufficient sleep of respondents

		Crude Model		Model 1^a^		Model 2^b^		Model 3^c^	
		PR	95%CI	PR	95%CI	PR	95%CI	PR	95%CI
Men	Lowest	1.47	(1.30–1.66)	1.22	(1.08–1.37)	1.24	(1.10–1.40)	1.22	(1.08–1.38)
	Low	1.44	(1.27–1.63)	1.18	(1.05–1.34)	1.21	(1.07–1.36)	1.20	(1.06–1.36)
	High	1.33	(1.17–1.51)	1.21	(1.07–1.36)	1.20	(1.07–1.35)	1.19	(1.06–1.34)
	Highest	Reference		Reference		Reference		Reference	
Women	Lowest	1.22	(1.10–1.36)	1.10	(1.00–1.22)	1.11	(1.00–1.22)	1.11	(1.00–1.22)
	Low	1.20	(1.08–1.34)	1.11	(1.00–1.22)	1.10	(1.00–1.22)	1.10	(1.00–1.22)
	High	1.17	(1.06–1.29)	1.09	(0.99–1.19)	1.08	(0.99–1.19)	1.08	(0.98–1.19)
	Highest	Reference		Reference		Reference		Reference	

^a^: Adjusted for age

^b^: Adjusted for age, education, occupation, and annual household income

^c^: Adjusted for age, education, occupation, annual household income, smoking, alcohol drinking, physical activity, BMI and presence of NCD

**Table 4:** Prevalence ratios of each neighborhood social capital dimension for insufficient sleep of respondents

	Crude Model			Model 1^a^		Model 2^b^		Model 3^c^	
	PR	95%CI		PR	95%CI	PR	95%CI	PR	95%CI
Feeling of fellowship									
Men	Lowest	1.36	(1.20–1.53)	1.17	(1.04–1.32)	1.21	(1.08–1.36)	1.19	(1.06–1.34)
	Low	1.43	(1.29–1.58)	1.19	(1.07–1.31)	1.22	(1.10–1.35)	1.21	(1.09–1.34)
	High	1.22	(1.09–1.36)	1.11	(1.00–1.23)	1.12	(1.01–1.24)	1.12	(1.01–1.24)
	Highest	Reference		Reference		Reference		Reference	
Women	Lowest	1.21	(1.09–1.35)	1.14	(1.03–1.26)	1.15	(1.04–1.27)	1.15	(1.04–1.27)
	Low	1.24	(1.14–1.36)	1.11	(1.02–1.21)	1.13	(1.03–1.23)	1.12	(1.03–1.22)
	High	1.17	(1.07–1.28)	1.08	(0.99–1.18)	1.08	(0.99–1.18)	1.08	(0.99–1.18)
	Highest	Reference		Reference		Reference		Reference	
Emotional Social Support									
Men	Lowest	1.50	(1.31–1.72)	1.29	(1.13–1.47)	1.29	(1.13–1.47)	1.28	(1.12–1.46)
	Low	1.40	(1.23–1.61)	1.26	(1.11–1.44)	1.26	(1.11–1.43)	1.26	(1.11–1.43)
	High	1.26	(1.09–1.47)	1.22	(1.06–1.41)	1.21	(1.05–1.40)	1.21	(1.05–1.39)
	Highest	Reference		Reference		Reference		Reference	
Women	Lowest	1.07	(0.97–1.18)	1.01	(0.92–1.11)	1.01	(0.92–1.10)	1.01	(0.92–1.10)
	Low	1.09	(0.99–1.20)	1.05	(0.96–1.15)	1.06	(0.97–1.15)	1.06	(0.97–1.15)
	High	1.05	(0.95–1.16)	1.01	(0.92–1.11)	1.00	(0.92–1.10)	1.00	(0.92–1.10)
	Highest	Reference		Reference		Reference		Reference	
Reciprocity norm									
Men	Lowest	1.08	(0.94–1.25)	1.00	(0.88–1.14)	1.04	(0.91–1.19)	1.02	(0.89–1.16)
	Low	1.25	(1.12–1.39)	1.10	(1.00–1.22)	1.15	(1.04–1.27)	1.14	(1.03–1.26)
	High	1.15	(1.03–1.29)	1.08	(0.96–1.20)	1.08	(0.97–1.20)	1.07	(0.96–1.19)
	Highest	Reference		Reference		Reference		Reference	
Women	Lowest	1.21	(1.07–1.36)	1.11	(0.99–1.25)	1.12	(1.00–1.25)	1.11	(0.99–1.24)
	Low	1.20	(1.09–1.32)	1.08	(0.99–1.19)	1.09	(0.99–1.19)	1.08	(0.99–1.18)
	High	1.17	(1.06–1.30)	1.11	(1.01–1.22)	1.10	(1.00–1.21)	1.09	(0.99–1.20)
	Highest	Reference		Reference		Reference		Reference	
Perceived neighborhood trust									
Men	Lowest	1.47	(1.25–1.74)	1.17	(1.00–1.37)	1.19	(1.02–1.39)	1.19	(1.02–1.38)
	Low	1.47	(1.26–1.71)	1.18	(1.02–1.36)	1.19	(1.03–1.37)	1.18	(1.03–1.36)
	High	1.34	(1.14–1.58)	1.17	(1.00–1.36)	1.18	(1.01–1.37)	1.17	(1.01–1.36)
	Highest	Reference		Reference		Reference		Reference	
Women	Lowest	1.35	(1.17–1.55)	1.13	(0.99–1.30)	1.14	(1.00–1.31)	1.13	(0.99–1.30)
	Low	1.39	(1.22–1.57)	1.15	(1.02–1.29)	1.15	(1.02–1.29)	1.14	(1.01–1.28)
	High	1.24	(1.09–1.42)	1.10	(0.96–1.25)	1.08	(0.96–1.23)	1.08	(0.95–1.23)
	Highest	Reference		Reference		Reference		Reference	
Social activity or co-operation									
Men	Lowest	1.44	(1.26–1.65)	1.22	(1.07–1.39)	1.24	(1.08–1.41)	1.20	(1.06–1.37)
	Low	1.38	(1.22–1.57)	1.21	(1.06–1.37)	1.23	(1.08–1.39)	1.21	(1.07–1.37)
	High	1.31	(1.14–1.51)	1.22	(1.07–1.39)	1.20	(1.05–1.37)	1.18	(1.03–1.34)
	Highest	Reference		Reference		Reference		Reference	
Women	Lowest	1.18	(1.05–1.32)	1.05	(0.94–1.17)	1.04	(0.93–1.16)	1.03	(0.93–1.15)
	Low	1.15	(1.03–1.28)	1.03	(0.93–1.14)	1.01	(0.92–1.12)	1.01	(0.91–1.12)
	High	1.14	(1.01–1.28)	1.03	(0.92–1.15)	1.02	(0.91–1.14)	1.01	(0.90–1.13)
	Highest	Reference		Reference		Reference		Reference	

^a^Adjusted for age

^b^Adjusted for age, education, occupation, and annual household income

^c^Adjusted for age, education, occupation, annual household income, smoking, alcohol drinking, physical activity, BMI and presence of NCD

## Discussion

Our findings demonstrated a significant relationship between NSC and sleep duration in men but not in women. In particular, NSC was negatively associated with insufficient sleep in the men even after controlling for all potential confounders. Each dimension of NSC except the feeling of fellowship, was also negatively related to insufficient sleep only in men. Furthermore, even in the working age group, the low NSC groups experienced the insufficient sleep more than the highest NSC group for both genders.

A few studies investigated the association between NSC and sleep duration by genders. The research by Toyosato and Takakura showed that through the sleep duration of women, the perceived trust of NSC was associated with self-rated health, and the authors proposed that NSC should be promoted for the improvement of sleep duration in Japanese women [8]. In contrary, our study found that low NSC was related to insufficient sleep in men but not in women. Although the reason for a discrepancy was unclear, results from both studies suggested there were gender differences in the relationship between NSC and sleep duration. Thus further prospective study is needed on the association between NSC and health behavior considering the gender pattern.

Another study in Finland demonstrated a positive association between NSC and adequate sleep of 7–8 h per night [7]. Our present findings are supported by that study, which specifically indicated that people with high levels of social participation and networks were likely to have healthier behaviors than those with low levels although the authors did not take account for the gender differences in the analysis. In contrast, Mohnen et al. [3] reported that they observed no association between NSC and sleeping habits. As the authors did not access a gender disparity in the analysis, it may lead to the different results from ours. Another explanation for the inconsistent results between the studies may be due to varying approaches of social capital [62]; while the authors assessed NSC using a collective approach, we used an individual approach. Furthermore, all of the studies mentioned above treated sleep duration as a mediator between NSC and health outcomes. However, we investigated the direct association considering other potential confounders, which were not fully examined in the previous studies. Moreover, we analyzed the association by different dimensions of NSC. These characteristics of our studies guarantee the robustness of our results.

A handful studies have described a direct relationship between NSC and sleep disturbances, which supports our findings. Takahashi et al. [9] reported that Japanese workers who had higher neighborhood or workplace social capital had a better quality and quantity of sleep. Their finding was similar to our results from subgroup analysis although the authors did not consider gender effects in the study. One of the reasons for the association between higher social capital and a better sleep among workers may be due to the barrier effect, where NSC acts as the shock absorber to the stress and strain of the workplace [63, 64]. Another possibility is that NSC relieves psychosocial or environmental stresses which disturb the sleep [65] via the mental wellbeing obtained from mutual trust, reciprocity, wider social networks and social supports [66, 67]. Moreover Bassett and Moore [10] argued that the lower neighborhood trust in a Canadian community increased the likelihood of restless sleep in women and that lower network social capital increased the likelihood of restless sleep in men. On the other hand, our results demonstrated that all dimensions of NSC were associated with insufficient sleep in men, but not in women. We speculate that social roles and responsibilities may shape sleep disturbances differently in men and women.

David-Barrett T et al. reported that men have larger social networks and higher thin trust each other compared to women [68]. In Japan, men are likely to spend longer time on active free-time activities, work and work-related activities than women [21]. Because of these activities outside, Japanese men may build social network from accumulating social norms or shared value through the expended loose social networks, which may relieve the distresses of sleep disturbance [12]. On the other hand, women in Japan accept gendered responsibility and have a desire to fulfill their social role as mother, spouse and caretaker for children and other family members [69]. In addition women generally tend to build emotional based, time-consuming kinship relationship among family members and close friends which creates fewer friendships or social networks with thick trust in women [68]. According to a report by statistics bureau in Japan [21], women tend to spend more time on communication with family members and thus they are likely to create thick trust and fewer social networks than men. Moreover Japanese women spend more time on child caring, care-giving and unpaid housework than men [21]. These responsibilities in women may relate to higher levels of depression, anxiety and affection, causing insufficient sleep. Another possibility is that hormonal effects during pregnancy, lactation, pre- and post-menstrual states, and/or pre- and post-menopause status in women induce sleep disturbances [38].

A number of explanations may account for the association between NSC and sleep duration. First, stressful conditions in a neighborhood contribute to alterations of the adaptive nature of sleep through psychosocial-cognitive pathways, because sleep has been recognized as largely psychological influential behavior [70, 71]. Second, living in a trustable community makes one feel safe and secure, which can contribute to falling asleep. Braithwaite [72] explained that unsafe, distrustful and highly stressed neighborhood conditions may relate to poor sleep. Third, a sense of membership in one’s community endows people with self-esteem, which acts as a social experience to alter health behavior [73]. Fourth, loosely knit relationships among residents adversely affect individuals’ psychological well-being, which is important for good sleep [74]. Finally, a neighborhood can give support for the distribution of information on healthy behaviors. A study in a Dutch community indicated that social support and information were potentially important for modifying health behaviors [75]. Although several mechanisms have been considered to underlie the association between NSC and sleep duration, the existence of a gender difference in that association was not clear until now.

Our study has several limitations. First, due to the study’s cross-sectional design, we were not able to analyze the cause-effect relationship between NSC and insufficient sleep. Thus, insufficient sleep itself may adversely affect both physical and mental well-being, which in turn has a negative impact on NSC. Future studies should examine the causal relationship in the gender gap between social capital and sleep duration. Second, the validity of NSC was not accessed and thus the conclusions should be drawn with a caution although we had advantages of imparting different dimensions of NSC. The development of validated questionnaires for NSC will be required in future researches for generalizability. Third, we evaluated the respondents’ subjective sleep duration as a main outcome variable using a self-reported questionnaire. Although we used self-reported sleep duration in our study, a previous study demonstrated that using self-reported results was as reliable as the objective assessments [76]. Thus a comprehensive approach including wrist actigraphy should be performed. In addition, evaluating sleep quality is important for sleep examinations, but we could not assess sleep quality as well as other complex disorders of sleep in this study. We also dichotomized the variable of sleep duration, and doing so could have under- or over-estimated the results. Forth, our respond rate of 63.2% was not much high so that it might lead to selection bias. Finally, we used several factors to control confounding effects, but other unmeasured possible confounders such as stress might affect the association between social capital and insufficient sleep.

## Conclusions

Our present findings demonstrated that having lower NSC was associated with insufficient sleep among Japanese adults, particularly among men. From a perspective of public health, social and environmental factors are modifiable and adaptable to healthier behaviors. Population-based studies of gender gap in NSC and sleep duration are worth conducting, toward the promotion of positive health-related behaviors. The context of NSC with gender differences should be considered when implementing interventions, strategies and policies to promote healthier behaviors regarding adequate amounts of sleep.

## Additional file

Additional file 1: Prevalence ratios of neighborhood social capital in working age group for insufficient sleep of respondents. (DOCX 30 kb)

## Abbreviations

BMI: Body mass index
CIs: Confidence intervals
FCS: Fully conditional specification
hrs: hours
MI: Multiple imputation
NCDs: Non-communicable diseases
NSC: Neighborhood social capital
OECD: Organization for Economic Co-operation and Development
*P*: *P*-value
PRs: Prevalence ratios
TOL: Tolerance value
VIF: Variance inflation factor
WHO: World Health Organization
