# Supplementary material for: Neighborhood social capital and sleep duration: a population based cross-sectional study in a rural Japanese town
Source: BMC Public Health. 2018 Mar 12;18:343. doi: 10.1186/s12889-018-5204-4 (PMC5848537; doi:10.1186/s12889-018-5204-4)
Supplement: Supplementary file 1 — Prevalence ratios of neighborhood social capital in working age group for insufficient sleep of respondents. (DOCX 30 kb) [file 12889_2018_5204_MOESM1_ESM.docx]

| **Additional file 1.** **Prevalence ratios of neighborhood social capital in working age group for insufficient sleep of respondents** | | | | | | | |
| --- | --- | --- | --- | --- | --- | --- | --- |
|  |  |  |  |  |  |  |  |
|  |  | **Crude Model** | | **Model 1^a^** | | **Model 2^b^** | |
|  |  | **PR** | **95%CI** | **PR** | **95%CI** | **PR** | **95%CI** |
| Men | Lowest | 1.47 | (1.30-1.66) | 1.39 | (1.23-1.56) | 1.34 | (1.19-1.51) |
|  | Low | 1.44 | (1.27-1.63) | 1.35 | (1.19-1.53) | 1.32 | (1.17-1.49) |
|  | High | 1.33 | (1.17-1.51) | 1.27 | (1.13-1.43) | 1.25 | (1.11-1.42) |
|  | Highest | Reference | | Reference | | Reference | |
| Women | Lowest | 1.22 | (1.10-1.36) | 1.15 | (1.04-1.28) | 1.14 | (1.03-1.27) |
|  | Low | 1.20 | (1.08-1.34) | 1.14 | (1.03-1.26) | 1.13 | (1.02-1.25) |
|  | High | 1.17 | (1.06-1.29) | 1.13 | (1.02-1.24) | 1.12 | (1.01-1.23) |
|  | Highest | Reference | | Reference | | Reference | |
|  |  |  |  |  |  |  |  |
| ^a^: Adjusted for age, education, occupation, and annual household income. | | | | | | |  |
| ^b^: Adjusted for age, education, occupation, annual household income, smoking, alcohol drinking, physical activity, BMI and presence of NCD. | | | | | | | |
